# COVID-19 Personal Protective Behaviors during Large Social Events: The Value of Behavioral Observations

**DOI:** 10.3390/bs14010063

**Published:** 2024-01-17

**Authors:** Ashley Gould, Lesley Lewis, Lowri Evans, Leanne Greening, Holly Howe-Davies, Jonathan West, Chris Roberts, John A. Parkinson

**Affiliations:** 1 Public Health Wales, Cardif CF10 4BZ, UK; 2 Somerset Council, Somerset TA1 4DY, UK; 3 Costain Group Ltd., Maidenhead SL6 4UB, UK; 4 School of Management, Swansea University, Swansea SA1 8EN, UK; 5 Welsh Government, Cardiff CF10 3NQ, UK; 6 Wales Centre for Behaviour Change, Department of Psychology, Bangor University, Bangor LL57 2AS, UK

**Keywords:** COVID-19, behavior, observation, mass-gathering, dual-process, prevention

## Abstract

Within the context of reopening society in the summer of 2021, as the UK moved away from ‘lockdowns’, the Government of Wales piloted the return of organized ‘mass gatherings’ of people at a number of test events. The current study reports behavioral observations that were made at two of the test events to inform this process. The researchers were particularly interested in four key factors: how (1) context within a venue, (2) environmental design, (3) staffing and social norms, and (4) time across an event, affected the personal protective behaviors of social distancing and face-covering use. Data collection was undertaken by trained observers. Adherence to protective behaviors was generally high, but there is clear evidence that these behaviors were shaped in a systematic way by the environment, situational cues, and the passage of time during the events. Some instances of large-scale non-adherence to personal protective behaviors were documented. An analysis within a dual-process framework suggests ways to understand and respond to supporting target health behaviors in groups of people where intervention is deemed valuable, such as in complex or ambiguous contexts. This is one of the first studies to include a ‘true’ behavioral measure in understanding human responses to COVID-19. It demonstrates that behavioral observations can add precision and granularity to understanding human behavior in complex real-world contexts. Given the significant physical and mental health burden created acutely and chronically by COVID-19, this work has implications for how governments and organizations support target populations in other complex challenges facing us today, such as in sustainability, and healthy lifestyle behaviors. An individual’s intentions are not always matched by their actions, and so the findings support a balanced liberal paternalistic approach where system-level changes support appropriate individual-level decisions to engender collective responsibility and action.

## 1. Introduction

Across the world, countries responded to Coronavirus (SARS-CoV-2) 2019 (henceforth ‘COVID-19’) by imposing a variety of measures to suppress transmission of the virus [1]. Along with infection control measures such as the development and implementation of a vaccine, non-pharmaceutical interventions were also employed. Whilst these measures and restrictions were designed to reduce contagion, they also had significant collateral impacts on physical and mental health across populations. As documented by Amsalem and colleagues [2], in a similar manner to previous large-scale disease outbreaks, COVID-19 produced significant negative impacts on mental health, but also engendered stigma and rejection both towards survivors of infection as well as to individuals from foreign countries that were significantly affected by the outbreak. Moreover, individuals working on the front line in response to COVID-19 also showed significant mental health impacts [3], including post-traumatic stress, burnout, and grief. Interestingly, a recent study [4] sought to understand how some individuals were resilient to these stressors and were able to adapt in a positive ‘growth’ manner (a term the authors called transilience). Of clear importance is understanding how such large-scale challenges impact mental health and behavior in order to be prepared for future population ‘shocks’.

A critical component of the human response to COVID-19 (as with previous outbreaks), was the role of behavior [5,6], and as countries cautiously began to open up their societies (during spring 2021 in the UK), there was a greater dependency on individual behavioral decision making in preventing COVID-19 spread [7]. Humans evolved as social animals and much of our behavior focuses on the development and maintenance of relationships. Indeed, there is evidence at behavioral and neuroscientific levels that we are ‘herd’ animals and derive a deep-rooted comfort and sense of security in group settings and group coherence [8]. Unfortunately, a key driving force in contagious disease is that transmission risk is greatest when people gather together. We do not need to look far back in human history to understand this lesson. Writing in *Science* in 1919, Major George Soper [9] noted the three key factors preventing society from overcoming the Spanish Flu: (1) humans are very much social animals, (2) there is a low level of risk literacy in the population, and (3) behavior is often unconsciously driven and prosocial, and hence a continuous risk to transmission (and see [5]). 

During the initial ‘hard’ lockdowns in the UK in 2020, regulation and punitive action served as key drivers of behavior and there was little requirement for individual evaluation of risk and choice. However, as societies opened up, the responsibility that was placed on individuals grew as social situations became more complex and appropriate behavior was more dynamic and context-dependent. In these circumstances, the importance of understanding the drivers of behavior became clear to policy makers, health providers, and local communities more generally, such that communications, policies, and interventions are designed to best support dynamic decision-making processes in complex and ambiguous situations. 

An underlying tenet in this approach is an acknowledgement that there are multiple drivers of human behavior [10,11,12,13] and hence an effective approach to infection control, such as with COVID-19, can only be realized by gathering and utilizing insights into those multiple key drivers. Additionally, COVID-19 infection control measures, by their nature, could be considered a cost to individuals but a benefit to society at large. As such, at an individual level, cost–benefit analyses will also shape intended behaviors [14,15] with respect to such factors as financial impact, effort, and opportunities. Data collected throughout the pandemic in Wales (https://www.gov.wales/survey-public-views-coronavirus-covid-19-29-april-2-may-2022, accessed on 4 November 2023) indicated that the majority of people self-reported social distancing, regular hand washing, and the use of face coverings where required [16]. However, other studies have indicated low levels of test-seeking behavior [17], with rates at certain times across the pandemic significantly lower than likely necessary for effective infection control. Whilst people have good intentions, these might not always be followed by good actions. Indeed, a recent review of the efficacy of lockdowns (or ‘stay-at-home’ orders) and other social-gathering regulations, acknowledged that adherence was a critical but understudied factor in efficacy [18]. Challenges to adherence have been evidenced by one of very few behavioral observational studies that looked at COVID-19 transmission [19]. Behavior in bars was observed between July and August 2020, during a relative easing of COVID-19-regulatory measures, and whilst bar owners put measures in place to enable COVID-19-safe activity, there were many recorded instances of non-adherent behavior. Whilst the use of questionnaires and self-report measures have been demonstrated to be effective in capturing insights into COVID-19 (e.g., mental health impacts; [2,3,20]), the current study focused selectively on the observational capture of behavior in order to provide a quantitative documentation of adherence.

One powerful, and seemingly unconscious, driver of human behavior is through social norms [8,14]. In selecting behaviors, individuals have a strong tendency to look to the behaviors of others, and in particular other individuals who share characteristics with them. Such social norm dynamics have been studied extensively [8,14,21,22] and can both explain human behavior in groups, as well as provide insight into how to influence an individual’s behavior in a chosen direction. 

The value of a ‘behavioral insights’ approach was originally recognized in observations that non-conscious behavioral drivers sometimes override intentions (such as social norms, habits, or contextual factors including physical architecture) as described above. As such, a failure to maintain personal protective behaviors during the COVID-19 pandemic, such as maintaining a two-meter distance, may reflect historical normative influences (i.e., how close people usually stand to have a conversation) even when there is a conscious intention to distance. This ‘intention–action gap’ is well recognized in the literature and is reflected in writing as far back as Plato’s *Republic*. A dual-process model posits two pathways vying for behavioral control (Table 1; [12,13]). Usually, these work in combination to support the direction of and energy for behavior. However, in certain circumstances, a difference in processing leads to conflict in behavioral control, such as normative influences, as described previously, or a cost–benefit analysis that leads to conflict between the different processes. Type 1 processes are likely to predominate (phylogenetically older, closer link in neural terms to behavioral control; [23]). Specific examples might include, for example, that individuals are not convinced by the dangers of COVID-19, or that individuals, intending to demonstrate individual toughness, might intentionally choose to not wear masks [15]. Alternatively, whilst an individual might have a strong intention to wear a mask, the prevailing social norm of reduced mask wearing, say in a group of inebriated individuals, might override the intention and lead to breaking the rules. 

The purpose of the current work was to apply such an approach to inform the Welsh Government’s ambition to reopen society, following the second lockdown between December 2020 and April 2021 [24]. Wales has a population of just over 3 million and would equate to a rural median-sized US state. The primary aim of the work was to gather behavioral insights at two pilot ‘mass gathering’ events in Wales, to complement other data, and help identify effective measures to reduce COVID-19-transmission risk with the prevailing (relatively low) population prevalence of COVID-19. 

### 1.1. Hypothesis

The overarching hypothesis was that, whilst individuals might intend to maintain protective behaviors at these events, the fact that they are less constrained (compared to ‘hard’ lockdowns) and in familiar settings (preponderance of old habits), might lead to behavior that is driven by automatic triggers (Type 1 processes) rather than prior intentions (Type 2 processes). These triggers could be internally elicited (e.g., by an existing cognitive schema of how to behave at a venue) or externally driven (e.g., responding to an activity during the event using social norms). The specific research questions reflected factors known to elicit automatic behaviors, as well as prior insights into COVID-19-related behaviors at similar events [5,19]. 

### 1.2. Research Questions

**RQ1:** To what extent did personal protective behaviors (social distancing and face-covering use) differ across different event contexts?
**RQ2:** To what extent did the physical environment impact personal protective behaviors?
**RQ3:** What role did staff play in influencing adherence to personal protective behaviors?
**RQ4:** To what extent did personal protective behaviors change over the duration of the event?

## 2. Materials and Methods

### 2.1. Design

This was an observational study of two events that were part of the piloting of ‘mass gathering’ events in Wales during the reopening following lockdown (Dec 2020–April 2021): Tafwyl Music Festival (15 May 2021) and a business conference at Celtic Manor Resort (20 May 2021). The study was conducted in accordance with the Declaration of Helsinki, and Ethical approval was obtained from Bangor University Research Ethics Committee. Informed consent was waived due to the observational nature of the research. Detailed risk assessments, approved by Public Health Wales’ Facilities, Estates and Compliance Lead, were conducted for both events to ensure the health and safety of the observers. 

The behaviors observed (dependent variables) were the COVID-19 personal protective behaviors of social distancing and the wearing of face coverings. For the current study, adherence to social distancing was defined as individuals maintaining a minimum of 2 m distance from each other. Observers followed the accepted approaches [19], and practiced making judgements about distance between individuals prior to the study. Adherence to mask wearing was defined as an individual wearing a mask (irrespective of design) which fully covered the mouth and nose (i.e., over the nose, under the chin) of the individual. The independent variables included the context within the venue (e.g., entry/exit, toilets, food/drink provision), environmental influences (e.g., signage, floor markings), staff and people (e.g., staff behaviors), time (e.g., changes in behavior across different time-points), and event management (e.g., cohorting of attendees). 

### 2.2. Context 

In Wales, during COVID-19 restrictions, events with large groups (>30 people) were restricted. As such, numerous events were cancelled or postponed. With the easing of restrictions in April 2021, the Welsh Government conducted pilot events across different sectors and of varying sizes, to inform guidance for future events. As gatherings with many people have the potential to be ‘super-spreader’ events and lead to outbreaks, it was important to understand how attendees may behave with regards to COVID-19 personal protective behaviors and how the event environment and organization could influence such behaviors. 

### 2.3. Participants

Participants were staff and members of the public attending the events. Approximately 500 people attended Tafwyl Festival and 80 people attended the Celtic Manor Conference. 

### 2.4. Materials

A semi-structured observation schedule was created to record quantitative counts and qualitative observations of behavior. Data were gathered discretely using Microsoft (MS) Forms, via observers’ smartphones, with each monitored episode uploaded separately. Observers took a portable charger to maintain power on the devices, and also used notebooks to record counts/observations/details as necessary before inputting data into the online MS Form. All observers wore fluid resistant surgical face masks, maintained social distancing, and had individual hand sanitizer.

### 2.5. Data Collection

The work followed established practices in behavioral science for observations in the field [19,25]. Furthermore, the research team worked closely with the Welsh Government and event organizers when planning the observations. In both events, key event organizing staff were informed of the role of observers. However, attendees and the majority of staff and volunteers were not told the nature of the observations. Observers travelled independently to the events and arrived at a pre-agreed location before the official start times to discuss with the event organizers where they would conduct the observations. Observers were not covert, though attendees were not explicitly made aware of the observers’ roles, in order to limit social desirability bias. No observers were approached by attendees or staff during the events, though an honest explanation would have been provided if requested. Observers worked in line with COVID-19 guidelines and had two 1 h briefing sessions prior to each event to ensure knowledge and consistency of procedures, data definitions, and collection methods. 

### 2.6. Tafwyl Festival

Four observers attended Tafwyl (an annual Welsh-language cultural festival), all of whom were employees of Public Health Wales and members of the Prevention and Behavior Change Cell. Observers attended for the full duration of the event, observing attendee arrival and exiting, working in (socially distanced) pairs to increase measurement reliability. Observer-pairs rotated through different settings (tables, toilets, food/drink settings) and repeated each rotation four times over the course of the event. Both observer-pairs conducted timed observations, lasting between 2 and 5 min for each of the dependent variables (i.e., social distancing and mask wearing), recording counts of the behaviors occurring (i.e., non-adherence, as defined above), partially occurring, or not occurring. Each observer independently inputted their counts via an MS Form. Between timed counts of compliance, observations around antecedents, behaviors, and consequences were recorded, particularly when breaches of personal protective behaviors were observed.

### 2.7. Celtic Manor Conference 

Three observers attended the conference (a professional business conference held at a hotel-based conference venue), including two of the observers who attended Tafwyl Festival and one other member of the Prevention and Behavior Change Cell in Public Health Wales. Observers attended for the full duration of the event. Observations with all three observers commenced with delegate arrivals and during refreshment breaks, while each observer conducted observations independently when conference break-out sessions occurred. Observations were recorded with the same methodology as the Tafwyl Festival. 

### 2.8. Analysis

Data were aggregated as counts for the variables in question. During observation, observers tallied on a piece of paper the number of individuals adhering, partially adhering, or not adhering to a behavior. Only one behavior was observed within a timed observation to ensure reliability. After the timed observation, observers compared counts, agreed on a final number if there were discrepancies (usually the average of the two), and entered data into the online form. Observers then started a new timed observation of a different behavior where appropriate. Qualitative data were themed according to the research questions. The results were sense-checked and discussed across the research team. 

## 3. Results

Each event was carefully planned to promote personal protective behaviors. At Tafwyl, attendees arrived in ‘bubbles’ and were required to remain at designated tables throughout the day. Attendees were not permitted to approach food and beverage vendors directly; all ordering was done via an online platform, with staff delivering food and beverages. Attendees’ movements were therefore limited, and none were observed approaching vendors. However, attendees were allowed to move around to visit restrooms, etc. 

At the conference, attendees were cohorted into three groups and provided with a colored wristband to indicate group membership. Observers recorded the establishment of group norms. Two groups were largely adherent throughout the event. However, one group contained a minority of attendees who frequently went against the advised behaviors. This persisted throughout the day and had a ripple effect on others in the group. 

Additionally, at the conference, observers recorded breaches in social distancing in queues for the staffed tea/coffee stations, though adherence tended to increase as delegates got closer to the stations. Lunch was a seated meal with food delivered by staff, designed to facilitate social distancing. There were no observed breaches during the meal. The majority of delegates left the dining area after they had finished eating, with a steady and managed flow, rather than a mass exit. 

### 3.1. RQ1. Did Personal Protective Behaviors (Social Distancing and Face-Covering Use) Differ across Different Event Contexts?

Observations indicated that personal protective behaviors differed across different contexts at both events (Table 2). At Tafwyl, adherence to social distancing was highest when attendees were sat at their pre-designated tables, reduced when at the toilet block, and was very low upon entry to and exit from the event. Similarly, at Celtic Manor, the lowest adherence was observed when moving between rooms and upon exiting the event. Full adherence to face-covering use at Tafwyl was lowest when queuing upon entry to and exiting the event, whereas at the conference there was full adherence across different situations. 

### 3.2. RQ2: How Did Environmental Features Impact Personal Protective Behaviors?

Some environmental interventions were implemented to facilitate the COVID-19-safe behaviors of attendees at the two events. At Tafwyl, screens displayed prompts to wear face coverings and social distance between artists’ performances and there were designated tables for groups to support maintaining a two-meter distance from other ‘bubbles’. Visible signage was limited and not in the immediate eye line of attendees for maximum impact. The noise level also prompted people to get closer together to be heard and remove face coverings.

Despite the large amounts of space available at Celtic Manor, breaches in social distancing were still evident. Although distance markers were placed on the floor at this event in the refreshments area, their visibility was limited due to their size and the non-contrasting carpet pattern. However, throughout the day, attendees were reminded to maintain social distancing, which, observers noted, prompted the majority of attendees to correct their behavior. Similarly, the environment at Tafwyl contained no floor markings in the toilet area, queues, and main walkways or bar areas to provide prompts for social distancing. 

At both events, hand sanitizer stations were placed at key points; however, observers noted that these were not used by the vast majority of people. Additionally, observers did not report any sanitizer refills and observed large amounts of sanitizer left at the end of the day. 

### 3.3. RQ3 How Did Staff Behavior Impact Personal Protective Behaviors?

Staff use of face coverings at Tafwyl was complete (100%, *n* = 48), with no breaches observed. However, the maintenance of social distancing was notably lower in staff than attendees. The majority of staff did not social distance, particularly bar staff (98%, *n* = 45). The area for collecting drink orders was very small and busy, which did not facilitate this behavior. Furthermore, during the staff briefing, observers noted that volunteer staff in particular were not social distancing, which was not rectified by the briefing lead. Throughout the event, observers reported volunteer staff breaching social distancing rules with other volunteers and attendees. Volunteers, on occasion, spent periods of time talking with attendees at the tables and sometimes removed their face coverings to talk to them, particularly when the music became loud. Additionally, the observers recorded a marked difference between the behavior of security staff in comparison to volunteers in terms of correcting breaches. Security guards identified breaches more quickly, and were quicker to intervene than volunteers, resulting in greater changes of attendee behaviors. Volunteers predominantly engaged in breaches of face-covering use and observations of intervention in social distancing behaviors were limited.

Similar trends were observed at Celtic Manor, with 75% of staff wearing face coverings during periods of observation. Prior to attendees arriving, it was estimated that around 20% of staff were maintaining social distance, increasing to 83% when attendees arrived. 

### 3.4. RQ4. Did Personal Protective Behaviors (Social Distancing, Face-Covering Use) Change over the Duration of the Event?

**Figure 1:**
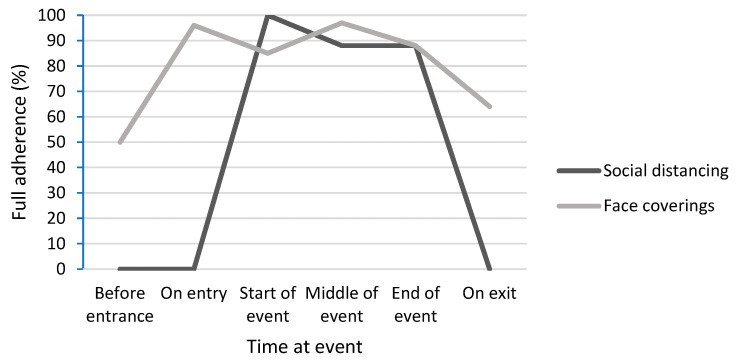
Adherence rates to social distancing and wearing face coverings over the duration of the Tafwyl event.

The observers reported no changes in personal protective behaviors over the duration of the conference event. However, at Tafwyl, adherence to social distancing and wearing face coverings was poorest at the start and end of the event (Figure 1). The most notable differences were with social distancing. Towards the end of the event, stewards were facilitating egress over a period of more than 30 min, where attendees became increasingly loud and disruptive. Stewards focused more on the disruptive attendees than the breaches of social distancing during this time. More frequent breaches occurred when attendees switched tables, which increased over time. 

## 4. Discussion

In general, adherence to COVID-19-protective behaviors by attendees across the two events was high, but with very clear indications that levels were shaped in a systematic way by the environment, situational cues, and the passage of time during the events (and see [26]). Notable across the events was the lack of social distancing by large numbers of people at points of access and egress, and before and after events. This was compounded (in terms of COVID-19 transmission risk) by the lack of face-covering protection in a notable minority, particularly at the point of leaving the Tafwyl event. Specific settings within events also led to the increased spatial density of attendees, such as queueing for toilets, and mass movement between different areas within each event.

These detailed behavioral observations highlight the importance of employing objective measures of behavior when evaluating interventions to support COVID-19-safe events. Furthermore, the systematic nature of non-adherence highlights the value of considering event coordination through a behavioral lens in order to understand and mitigate risk effectively, as indicated by Drury and colleagues [27]. 

For example, cohorting/batching attendees with managerial and environmental controls appeared to help in managing crowding (and see [28]). More generally, environmental and service restructuring, including seating plans, floor markings, food and beverage delivery (and remote ordering), and physical barriers/pathways, worked well to shape the behaviors of large numbers of individuals. There was evidence that live communications at events to remind people of requirements and positively acknowledge adherence, also served to ‘reset’ behaviors and improve adherence. There was some evidence of group norms reinforcing both pro-social and less helpful behavior [29]. In a recent review of ‘nudges’ in healthcare contexts, it was found that environmental adaptations and the selection of default behaviors were most effective in changing behavior [30]. This aligns with the current study, where a reshaping of the environment appeared to be particularly effective in promoting personal protective behaviors. Likewise, Chater and Loewenstein [31] have argued for a balance of behavior change interventions between those focusing on the decision-making burden resting predominantly on the individual, and changes at a larger system level which regulate the target behavior. In this case, the design of the environment, with a focus on facilitating personal protective behaviors, played a key role in ensuring target behaviors were carried out [32]. 

There was clear value, but variability, in staff intervening in attendee behaviors. Staff and volunteers acted as role models and provided explicit guidance around appropriate behavior (offering advice, correcting failures of adherence). However, there was behavioral evidence of ‘perceived immunity to risk’ in some staff groups, particularly volunteers. A sustained/widespread lack of social distancing, and the removal of face coverings (to aid communication), was observed in these groups. Given the high number of contacts some staff have, adherence to personal protective behaviors in this group is cause for concern. Clarity on role definition and training around advising in ways that sustain safe behaviors, and systems to maintain that throughout the event, would help increase adherence. A novel finding was that adherence waned with the passage of time across the events. There are a number of factors that might account for this: increased alcohol consumption at one of the test events; fatigue in inhibitory control; habituation; and reduced anxiety as a novel context becomes familiar. This temporal factor is not well understood and would benefit from further investigation. Overall, behavioral adherence broke down under conditions where staff were not present, there was a lack of environmental design/ signaling (including physical interventions, service design, cohorting or communications), and later in the event where the situation was less constrained and individuals were apparently less cognitively vigilant, i.e., where there were fewer external cues to promote appropriate behavior and cognitive control in individuals.

Social norms can produce a strong behavioral influence [8,14] and some of the observed breakdowns in adherence to protective behaviors may well have been due to such factors. For example, the ‘crowding’ at ingress and egress, and lack of mask wearing towards the end of the Tafwyl event, may reflect social norms. One avenue for future investigation may be in exploring threshold models of behavior and the role of ‘tipping points’, i.e., the proportion of a group needed to carry out a new behavior to then generate a new and significant influence on the behavior of others [21,22]. By integrating measures that might interrogate both Type 1 and Type 2 processes, future work can help disentangle the contributions of these different underlying processes. 

Taken as a whole, one interpretation of these data is that individuals at the events were using external stimuli and signals (including norms) to make decisions and control behaviors, rather than dynamically and rationally attempting to evaluate risk themselves in the moment and then act accordingly [11,12,13]. An important tactic, then, for uncertain or ambiguous contexts, may be to focus efforts to provide, and maintain, such cues at events. Where these were present, in the current study, adherence levels were higher. Linked to this, where settings are inherently less structured and transmission-risk is increased, there is utility in providing frequent communications, alongside steward presence/action (which might include role-modelling of normative expectations), to support increased reflective decision-making.

One area where future work could build upon the current study is in developing the behavioral methodology to capture a richer dataset and provide a more comprehensive analysis. Whilst the current work provided a clear descriptive picture of individual and group behavior, the motives or drivers of this behavior could not be diagnosed. On the one hand, questionnaires and other self-report data from individuals present at the events could provide additional insight in target situations [20,33], e.g., where adherence to protective behavior broke down, or where social norms emerged that could not easily be explained. Likewise, the use of video, perhaps even with some ‘artificial intelligence’ support, might allow for a more precise understanding of behaviors and their proximal triggers. Furthermore, approaches such as using threshold modelling of collective behavior could be integrated to better understand normative influences in these complex scenarios [21,22]. 

**Figure 2:**
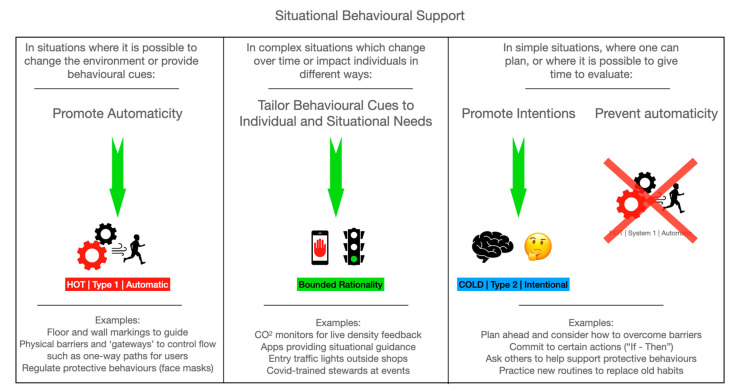
Situational strategies to support COVID-19-safe behaviors.

From the perspective of dual-process theory [10], behavior change should be most effective when (1) Type 1 processes which automatically trigger incorrect behaviors are suppressed, allowing Type 2 cognitive processes to identify a correct behavioral response; (2) Type 1 processes are facilitated when they are likely to automatically trigger the correct target; and finally, (3) context-specific active decision-making support (perhaps through digital tech) would enable appropriate behaviors to be identified and triggered in dynamic situations where contingencies are changing rapidly. Figure 2 schematizes this approach to proactive support for behavior change.

The behavioral observational results of the current study provide examples of this approach in action and point to some key areas of focus in supporting personal protective behaviors in times of ambiguity or uncertainty. Firstly, social expectations of behavior seems to be an important precursor to event management. It is important to give clear communications about the behavioral expectations before, during, and after events (suppress T1, empower T2). Furthermore, the supported maintenance of the behaviors (announcements, active staffing) and priming of clients/guests before arrival (e.g., mental models to follow) would be beneficial. Secondly, regarding crunch-points and flow, the movement of individuals and groups at an event could be modelled and prototyped by organizers and included in event planning. The use of a ‘service design’ approach might reduce the risk of bottlenecks and bunching by optimizing appropriate T1 processes [32,34]. Thirdly, consistent staff training and practices during events appeared to be critical in the current work. This includes methods to support adherence such as use of role-modelling and reinforcing expectations around appropriate behaviors (optimizing T1). In the current work, some staff ignored personal protective behaviors through an apparent sense of invulnerability. In doing their job, staff are likely to interact with many more people at an event; therefore, they are at high risk and are more likely to have the potential to seed transmission and super-spreading. Future work looking at individual differences in response to risk communication would be valuable. Finally, environmental adaptation and design supported protective behaviors. The use of environmental triggers should be optimized (e.g., floor markings, client flows, batched entry/exiting) as external signals are likely the primary drivers of dynamic behavior at events, where people ‘fall back’ on pre-COVID-19 habits (optimizing T1 processes, and potentially instilling new habits; [35]). In unstructured areas, clear communications (including stewarding) and signage can aid reflective risk evaluation and behavioral decisions (suppress T1 and empower T2). These final changes might fall within the scope of system-level changes, as described by Chater and Loewenstein [31], in so far as designing the system (the rules of the game) so that the target behaviors become default are ‘baked in’ through physical barriers. With regard to personal freedoms, this approach aligns well with balancing intervention rigidity and control, which is described in the Nuffield ladder framework [36]. Where risk is highest, or consequences most dire, it is likely that restricting or regulating behavior will be more effective, employing Type 1 automatic processes.

From a broader perspective, the systematic breakdown in personal protective behaviors observed in the current study would likely have led to the increased spread of COVID-19. Indeed, in other work, we have begun to integrate measures of both behavior and of COVID-19 infection status [37] to provide a more sophisticated convergent approach to infection control. Nevertheless, large events present an increased risk for COVID-19 transmission (and other large outbreaks) and their costs and benefits should always be considered in the context of background levels of disease and the vulnerability of attendees. As noted in the introduction, whilst the physical health burden of COVID-19 is well documented, the mental health impacts, and other indirect traumas, are emerging as significant lasting costs to individuals and society [2,3,33,38], including stress, grief, burnout, depression, and suicide. These impacts appear to have an unequal impact across countries and demographics [39,40] and reinforce the need to understand the ways in which health sectors can treat mental health impacts, as well as better understand individual and social resilience to trauma [4,41] in preparation for future challenges and global shocks.

## 5. Conclusions

The significant negative impacts of COVID-19 have included physical and mental health and could be considered akin to post-traumatic stress disorder in their severity [33,42]. Finding effective ways to reduce transmission across differing levels of disease prevalence is critical in supporting future population health. Bavel et al. [5] conclude their article on the role of behavioral science in COVID-19 control by noting that, following the Spanish Flu pandemic, a paper published in *Science* [9] gave three key factors as barriers to prevention: poor risk literacy in the population, humans as social animals, and the fact that behavior is often unconsciously driven and hence a continuous risk to transmission. Our observational findings reported herein are consistent with these messages and perhaps reflect an ongoing lack of appreciation as to the utility of behavioral science for effective policy and practice. Figure 2 provides a schematic illustration of one approach to behavior change in different contexts and is built upon a richer understanding of the key drivers of behavior. Investment in building capacity in behavioral science in government (and partners) would be an important facilitator for the routine development of policies, interventions, and communications campaigns that are behaviorally informed at an early stage and aim to close the intention–action gap in population behaviors that are important for the health and prosperity of citizens and societies. Toolkits are available to facilitate this process, with the Behavior Change Wheel (incorporating COM-B; [43]), EAST [44], and MINDSPACE [45], along with the development of government and public sector toolkits, for example, by the World Health Organization (https://www.who.int/europe/publications/i/item/9789289058919, accessed on 4 November 2023) and by Public Health Wales: https://phwwhocc.co.uk/bsu/resources/, accessed on 4 November 2023). 

The current work resonates with Soper’s [9] ‘Spanish Flu’ analysis—that humans generally show poor risk literacy and that their behavior is often driven by unconscious, automatic cues which put them in greater danger of disease transmission. This provides clear direction for our preparedness for future infection control challenges, and other situations where mass behavior change is required, particularly where individuals might be expected to make dynamic behavioral decisions based on changing situational factors. In these scenarios, Figure 2 provides a framework which balances individual responsibility in decision-making with adaptations to overarching systems and environments to make the ‘safe’ behavior the easiest, or most likely. Without additional support, it is likely that pre-existing habits and intuitive responses, as well as social cues such as norms, will predominate [29,30,31,32,33,34,35].

The Royal Society recently conducted a series of reviews looking at the efficacy of non-pharmaceutical interventions during COVID-19, which suggested that social distancing measures can be highly effective in mitigating the risk of contagion within a population [18]. Environmental controls may also be advantageous, though more focused research is required in that regard [28]. A separate evaluation on the efficacy of communication strategies to promote personal protective behaviors indicated that government-level communications have generally been effective [46]. However, a notable limitation in many of the studies reviewed was the lack of the direct measurement of observable behavior in real-world settings. While the existing reviews underscored the effectiveness of interventions at the population or workplace level, they often lacked the granularity necessary for understanding nuances in individual behavior, and hence in designing subsequent interventions. A critical avenue for future research would be to emphasize the quantification of behavior as the dependent variable in empirical studies, to gain deeper insights into the systematic influences on behavior within these contexts.

## Figures and Tables

**Table 1:** Dual-process approach to behavior.

Type 1 Processes—HOT—Intuitive	Type 2 Processes—COLD—Cognitive
Older system, more direct control over behavior	Newer system in evolutionary terms
Reflexive, fast, automatically triggered	Reflective, slow, intentionally triggered
Easy, default, habitual, normative	Effortful, requires planning, limited capacity
Associative	Cognitive

**Table 2:** Adherence to personal protective behaviors across different situations. Observations/counts converted to % adherence for each situation. ‘Partial adherence’ is defined as social distancing of less than 2 m but more than 0 m; and for face covering, use in a non-optimal way e.g., worn under the nose, etc.

Behavior/Situation	Full Adherence (%)	Partial Adherence (%)	No Adherence (%)
**Tafwyl ^a^**			
*Social distancing*			
Queuing on entry	0	-	100
Entry	0	-	100
Tables	91	4	5
Toilets	49	35	16
Exit	0	-	100
*Face Coverings*			
Queuing on entry	50	-	50
Entry	99	-	1
Toilets	89	-	11
Exit	63	10	27
**Celtic Manor**			
*Social distancing*			
Waiting for conference to start	77	20	3
Moving between rooms	18	37	45
Lunch	95	5	0
Tea/coffee breaks	77	21	2
Listening to speakers	93	5	2
Exit	20	20	60
*Face coverings*			
Entry	100	0	0
Moving between rooms	100	0	0

^a^ The total N for each situation differs due to different observations in each context.

## Data Availability

Data are available from Public Health Wales. Please contact the corresponding author.
